# Site-specific processing of Ras and Rap1 Switch I by a MARTX toxin effector domain

**DOI:** 10.1038/ncomms8396

**Published:** 2015-06-08

**Authors:** Irena Antic, Marco Biancucci, Yueming Zhu, David R. Gius, Karla J. F. Satchell

**Affiliations:** 1 Department of Microbiology-Immunology, Feinberg School of Medicine, Northwestern University, 303 East Chicago Avenue, Ward 6-225, Chicago, Illinois 60611, USA; 2 Department of Radiation Oncology and Pharmacology, Feinberg School of Medicine, Northwestern University, 303 East Superior Avenue, Lurie 3-119, Chicago, Illinois 60611, USA

## Abstract

Ras (Rat sarcoma) protein is a central regulator of cell growth and proliferation. Mutations in the *RAS* gene are known to occur in human cancers and have been shown to contribute to carcinogenesis. In this study, we show that the multifunctional-autoprocessing repeats-in-toxin (MARTX) toxin-effector domain DUF5_Vv_ from *Vibrio vulnificus* to be a site-specific endopeptidase that cleaves within the Switch 1 region of Ras and Rap1. DUF5_Vv_ processing of Ras, which occurs both biochemically and in mammalian cell culture, inactivates ERK1/2, thereby inhibiting cell proliferation. The ability to cleave Ras and Rap1 is shared by DUF5_Vv_ homologues found in other bacteria. In addition, DUF5_Vv_ can cleave all Ras isoforms and KRas with mutations commonly implicated in malignancies. Therefore, we speculate that this new family of Ras/Rap1-specific endopeptidases (RRSPs) has potential to inactivate both wild-type and mutant Ras proteins expressed in malignancies.

## Abstract (web summary)

*V. vulnificus*, a bacteria that cause life-threatening septicaemia following wound infections or tainted food consumption, utilizes MARTX toxins for toxic effector delivery. Here the authors show that the MARTX virulence factor DUF5 targets the cellular MAP kinase pathway as a Ras and Rap1 site-specific protease.

## Graphical abstract

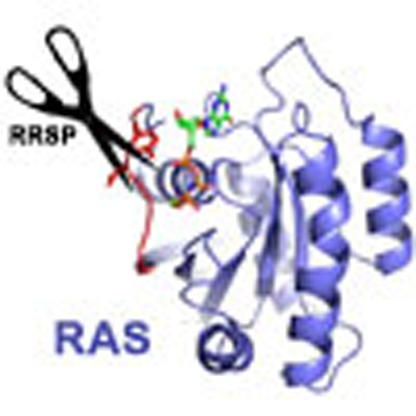

Rat sarcoma (Ras) oncoprotein is a small GTPase ubiquitous in eukaryotic cells and is a critical node that coordinates incoming signals and subsequently activates downstream target proteins. These targets include rapidly accelerated fibrosarcoma kinase (Raf), phosphatidylinositol-4,5-bisphosphate 3-kinase and mitogen-activated protein kinase (MAPK), which ultimately induces expression of genes directing cell proliferation, differentiation and survival. Regulation of Ras enzymatic activity is achieved by cycling between an inactive (GDP-bound) state and an active (GTP-bound) state. On activation, conformational changes in the Ras protein structure trigger Ras downstream signalling cascades by binding specific protein effectors^1^,^2^,^3^,^4^. Mutations in Ras proto-oncogenes are found in 9–30% of all human malignancies. In addition, Ras point mutations, which are observed at residues G12 and G13 in the P-loop and at Q61 in the Switch II region, are the most common mutations in human malignancies and are present in 98% of pancreatic ductal adenocarcinomas, 53% of colorectal adenocarcinomas and 32% of lung adenocarcinomas^5^,^6^,^7^. However, effective targeting of Ras has been very difficult and is considered a critical roadblock on the path towards generating new therapeutics against intractable human cancers^8^,^9^,^10^,^11^,^12^. Despite the potential of Ras proteins as therapeutic targets, there are no inhibitors for any of the three main human isoforms—HRas, KRas and NRas—or their constitutively activated mutant forms^8^,^9^,^10^,^11^.

From a microbial pathogenesis perspective, activation of Ras is central to cellular detection of bacterial lipopolysaccharide (LPS) and other pathogen-associated molecular patterns resulting in activation of innate immune defenses^13^. Although several bacterial toxins are known to target Ras by posttranslational modification to circumvent this important host response to infection, to date none have been shown to be highly specific for Ras^14^,^15^,^16^.

Multifunctional-autoprocessing repeats-in-toxin (MARTX) toxins proteins are large composite-secreted bacterial protein toxins that translocate across the eukaryotic cell plasma membrane and deliver multiple cytopathic and cytotoxic effector proteins from a single holotoxin by autoprocessing^17^,^18^. In our previous work, we showed that the most highly virulent strains of the sepsis-causing pathogen *V. vulnificus* produce a 5,206-amino acid (aa) MARTX toxin with an extra effector domain termed DUF5_Vv_, for the domain of unknown function in the 5th position^19^. In fact, bacterial strains that produce a MARTX toxin with DUF5_Vv_ are found to be 10- to 50-fold more virulent in mice than strains that produce a MARTX toxin without DUF5_Vv_ (ref. 19). These data directly connect DUF5_Vv_ with increased virulence during infection.

The 509-aa DUF5_Vv_ effector domain of the MARTX toxin was highly cytotoxic when ectopically expressed as a fusion to green fluorescent protein (GFP), resulting in rounding and shrinkage of cells^20^. Structural and functional bioinformatics studies have demonstrated that DUF5_Vv_ is comprised of two subdomains^20^,^21^. The amino-terminal C1 subdomain is a four-helix bundle that mediates localization to the plasma membrane by binding anionic phospholipids^21^,^22^. The carboxy-terminal C2 subdomain confers the cell rounding activity^20^. Moreover, DUF5_Vv_-C2 was found to inhibit growth when conditionally overexpressed in *Saccharomyces cerevisiae*^20^.

In this study, we used a combination of genetic, cell biological and biochemical strategies to probe the mechanism of action of the C2 subdomain, to understand the connection of DUF5_Vv_ to both cytotoxicity and increased virulence of the pathogen. We found that DUF5_Vv_ site-specifically processes both Ras and the closely related small GTPase Rap1. Both proteins are critical for activation of the innate immune response during infection, which explains the crucial role of this effector domain in the increased virulence of *V. vulnificus* strains that have DUF5_Vv_. As Ras is also important for cell proliferation in carcinogenesis, this enzyme could potentially be developed as a treatment for various types of tumours.

## Results

### DUF5_Vv_ causes ERK1/2 dephosphorylation

**Figure 1:**
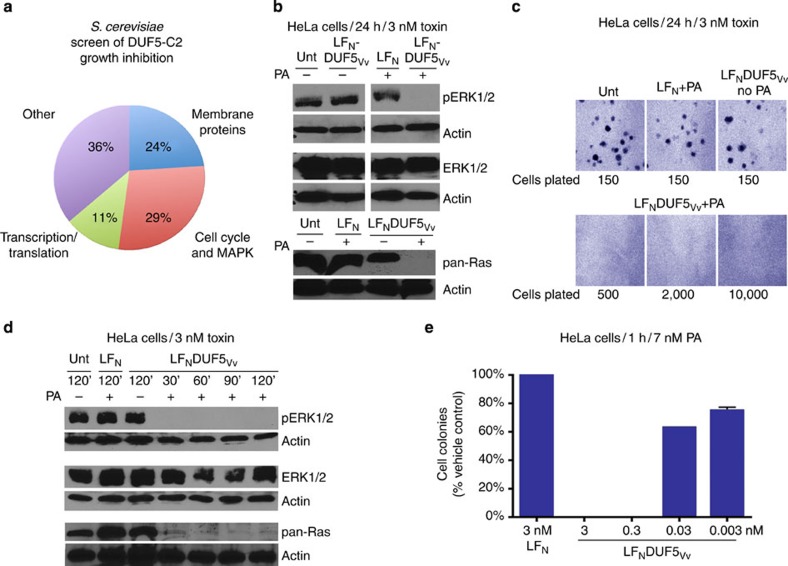
DUF5_Vv_-dependent disruption of Ras-ERK-dependent cell proliferation. (**a**) Major categories of yeast mutants enabling growth in the presence of DUF5_Vv_-C2. (**b**,**d**) Representative immunoblots (*n*=3) of lysates prepared from cells treated for 24 h (**b**) or time indicated (**d**) with LF_N_DUF5_Vv_ in the absence (−) or the presence (+) of PA. Trimmed ERK1/2 blots are shown unedited in Supplementary Fig. 2. (**c**,**e**) Clonogenic colony-formation assay (*n*=2) of cells treated for 24 (**c**) or 1 h (**e**). Error bars represent the range of the data.

Previously we showed that DUF5_Vv_-C2 is cytotoxic when ectopically expressed in eukaryotic cells^20^. As a strategy to identify molecular targets accounting for this cytotoxicity^20^, a genome-wide, arrayed, non-essential gene deletion library was screened for yeast strains that survived enforced expression of C2 (Supplementary Fig. 1). Of 4,709 yeast strains screened, 3.6% formed colonies on plates containing the inducer galactose, indicating that the yeast gene disruption suppressed C2-dependent growth inhibition. The hits were categorized based on information in the *Saccharomyces* Genome Database^23^. Eleven per cent of the mutant yeast strains that overcame growth inhibition due to DUF5_Vv_-C2 expression harboured deletions in genes for transcription and/or translation. These mutations probably reduce DUF5_Vv_-C2 expression, accounting for suppression of growth inhibition. Twenty-four per cent of the recovered yeast strains had defects affecting membrane or membrane proteins, possibly causing suppression of cytotoxicity due to the absence of the cellular target at the membrane (Fig. 1a).

Among the remaining hits, nearly half were connected to MAPKs or processes they regulate. Therefore, it was postulated that mammalian MAPK p38 and ERK1/2 could have altered activity during exposure of cells to DUF5_Vv_. We have previously demonstrated that the cytotoxic activity of DUF5_Vv_ can be isolated away from the large MARTX by fusing DUF5_Vv_ to the N terminus of anthrax toxin lethal factor (LF_N_DUF5_Vv_) and subsequently delivering the fusion protein to cells in culture using anthrax toxin protective antigen (PA)^20^. Therefore, we used this system to test for changes in MAPK signalling dependent on exposure of cells to DUF5_Vv_.

HeLa cervical carcinoma cells constitutively produce high levels of phospho-p38 and phospho-ERK1/2 (pERK1/2), making these cells an ideal model system to determine the underlying mechanism by which DUF5_Vv_ interferes with MAPK signalling (Supplementary Fig. 2). For cells intoxicated with LF_N_DUF5_Vv_ in combination with PA for 24 h, no change in levels of phospho-p38 was observed (Supplementary Fig. 2a). However, there was a marked absence of pERK1/2 in HeLa cells treated with LF_N_DUF5_Vv_+PA (Fig. 1b and Supplementary Fig. 2a). In addition, the first 276 aa of DUF5_Vv_, corresponding to the C1 membrane-targeting subdomain and the first 186 of C2 (C1C2A_Vv_), were sufficient to reduce pERK1/2 levels (Supplementary Fig. 2b), consistent with previous results showing that C1C2A_Vv_ is sufficient for cell rounding activity^20^. Thus, the yeast screen and subsequent studies in HeLa cells revealed that DUF5_Vv_ modulates the activation state of ERK1/2 without affecting p38.

### Ras depletion by DUF5_Vv_ inhibits cell division

Owing to its C1 membrane-targeting subdomain, DUF5_Vv_ is exclusively present at the plasma membrane^21^; hence, inactivation of membrane-localized Ras GTPases that control activation of ERK1/2 seemed a plausible mechanism for DUF5_Vv_-dependent ERK1/2 dephosphorylation^24^,^25^. Active Ras (GTP-bound) was probed using a G-LISA assay, where wells are coated with a Ras GTP-binding protein domain. Surprisingly, active Ras was undetectable in cell lysates intoxicated with LF_N_DUF5_Vv_+PA, suggesting that Ras was exclusively in the inactive, GDP-bound state (Supplementary Fig. 3). This result initially suggested that DUF5_Vv_ affects levels of active Ras-GTP. However, additional control experiments revealed that Ras protein itself was undetectable in cell lysates, as measured by immunoblotting with a monoclonal anti-RAS10 antibody that detects all isoforms of Ras^26^, including KRas, HRas and NRas (Fig. 1b). This experiment shows that DUF5_Vv_ directly targets the Ras protein rather than indirectly affecting its regulation.

If Ras and pERK1/2 are truly absent from DUF5_Vv_-treated cells, proliferation should be inhibited in intoxicated samples. To measure disruption in cell proliferation due to the inhibition of the Ras-ERK pathway, the toxin was removed by washing, and treated cells were plated and resulting colonies counted after a 14-day incubation period. HeLa cells intoxicated for 24 h did not produce colonies even when plated at almost 70-fold higher seeding densities than control-treated cells (Fig. 1c). Examination of ERK1/2 and Ras inactivation over time revealed that exposure of cells to 3 nM LF_N_DUF5_Vv_ for only 30 min was sufficient for nearly 100% inactivation (Fig. 1d and Supplementary Fig. 4). In addition, exposure of cells to LF_N_DUF5_Vv_ concentrations as low as 30 pM for 1 h was sufficient to significantly decrease cell proliferation (Fig. 1e). Overall, these studies reveal that DUF5_Vv_ directly targets Ras, resulting in loss of ERK1/2 phosphorylation and cell proliferation.

### Ras is cleaved at the N terminus in DUF5_Vv_-treated cells

**Figure 2:**
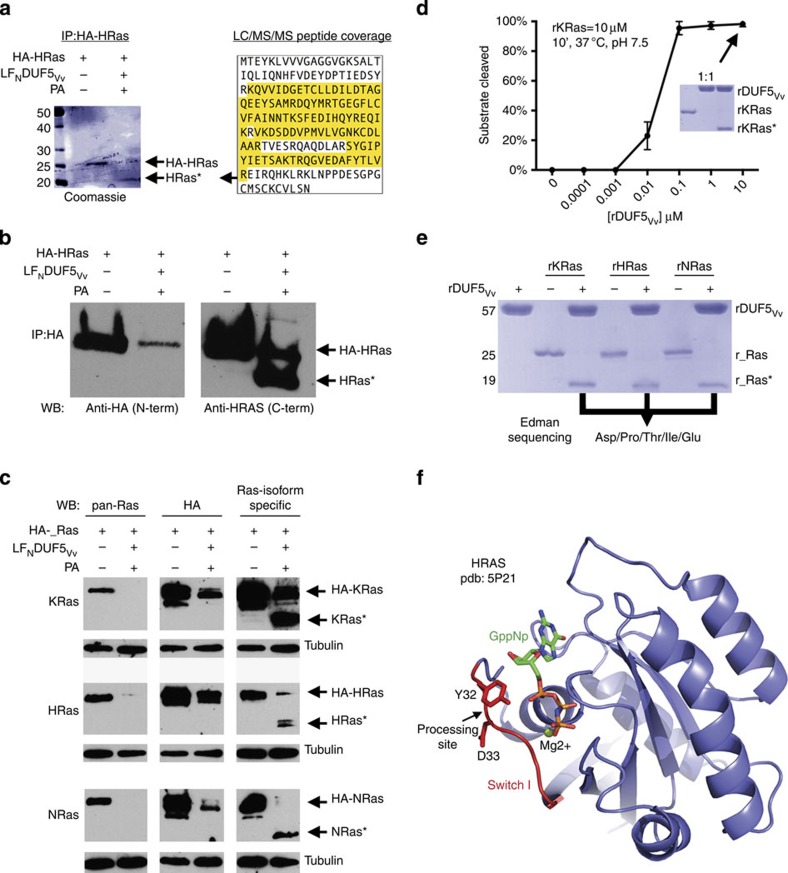
DUF5_Vv_ is a Ras site-specific endopeptidase. (**a**) Coomassie-stained 18% SDS-polyacrylamide gel of anti-HA immunoprecipitated proteins from cells expressing HA-HRas treated for 24 h as indicated. Lower band (HRas*) was excised for peptide sequencing with HRas peptide coverage highlighted in yellow. (**b**) Same fractions probed by immunoblotting to detect the N terminus (anti-HA) and C terminus (isotype-specific antibody). (**c**) Lysates from cells expressing HA-tagged KRas, NRas or HRas probed by immunoblotting as indicated. (**d**) *In-vitro* cleavage of 10 μM rKRas to KRas* with 10 μM rDUF5_Vv_ (inset) or concentration indicated. Error bars indicate mean±s.d. (*n*=3). (**e**) *In-vitro* cleavage of 10 μM rKRas, rHRas and rNRas with 10 μM rDUF5_Vv_. Identical results of Edman degradation were obtained for all three proteins. (**f**). Black arrow indicates the cleavage site in the Switch I region (red) of HRas^69^.

Only a few bacteria are known to specifically target Ras as a strategy to circumvent the host response and all do so by covalent attachment of nucleotide-sugar moieties to critical residues^14^,^15^,^16^. To investigate whether the loss of detectable Ras protein levels was due to proteolysis and/or a posttranslational modification that would mask the antibody epitope, HeLa cells were transfected to ectopically express HRas with a haemagglutinin (HA)-tag on the N terminus (HA-HRas), so as to facilitate immunoprecipitation with anti-HA antibody-coupled beads. Analysis of proteins immunoprecipitated from LF_N_DUF5_Vv_+PA intoxicated cells revealed a Coomassie-stained band with a molecular weight ∼5 kDa smaller than the band observed in the untreated cells (Fig. 2a left panel). Liquid chromatography–tandem mass spectrometry sequencing of tryptic peptides identified this protein as HRas, with no detection of the first three expected N-terminal peptides (Fig. 2a right panel).

When the elution fraction was probed with anti-HA or anti-RAS10 monoclonal antibodies that detect the N terminus, a quantitative loss of the full-length protein from intoxicated cells was observed (Fig. 2b, left panel). By contrast, an isoform-specific polyclonal antibody that detects the C terminus of HRas identified two bands of HRas: one representing the full-length HA-HRas and one ∼5 kDa smaller. We speculate this cleaved form of HRas was present in the immunoprecipation despite lacking the HA tag, because the HA-tagged fragment remained associated with the larger C-terminal fragment in the folded protein. This experiment suggested that DUF5_Vv_ induces clipping of Ras within the N terminus of the protein.

To verify that Ras is processed and to determine which isoforms of Ras are affected, cells were transfected to express HA-tagged KRas, NRas or HRas. In cells treated with LF_N_DUF5_Vv_+PA, western blot analysis of whole-cell lysates showed that all three isoforms were cleaved at the N terminus. The anti-HA and RAS10 monoclonal antibodies directed against the N terminus did not detect KRas, NRas or HRas in treated cells, whereas isotype-specific antibodies directed against the C terminus detected the smaller processed forms (Fig. 2c). A reduction in the total protein detected by the isoform-specific antibodies was also observed. This suggests that subsequent to processing, the cleaved forms are degraded, especially for HA-NRas and HA-HRas. These data show that Ras isoforms are not modified by addition of moieties but are instead severed near the N terminus, which is a novel mechanism for Ras inactivation.

### Recombinant DUF5_Vv_ can process all Ras isoforms *in-vitro*

Two possible explanations of our results are that DUF5_Vv_ activates a previously unknown cellular peptidase or functions as a Ras endopeptidase itself. To distinguish whether DUF5_Vv_ directly catalyses proteolytic processing of Ras, recombinant 6xHis-tagged DUF5_Vv_ (rDUF5_Vv_) and Ras isoforms (r_Ras) were expressed in *Escherichia coli* and purified. When mixed together for an *in-vitro* reaction, rKRas was efficiently cleaved within 10 min in a concentration-dependent manner (Fig. 2d). This reaction did not require addition of any other proteins or co-factors. rHRas and rNRas were likewise efficiently processed by purified rDUF5_Vv_ (Fig. 2e).

N-terminal sequencing of KRas, HRas and NRas cleaved products revealed that all Ras isoforms were identically cleaved between Y32 and D33 (Fig. 2f). These amino acids are found within the Ras Switch I region. Processing at this site would be expected to entirely abolish Ras signalling, as Y32 is required to orient and stabilize Switch I in the active (GTP-bound) state^27^. Cleavage within the Switch I region would further prevent the activation of downstream signalling cascades by disrupting the Ras effector protein interactions, thereby inhibiting activation of the ERK1/2 transcriptional regulator and decreasing cell proliferation^28^,^29^,^30^.

### Other DUF5 homologues cleave Ras

**Figure 3:**
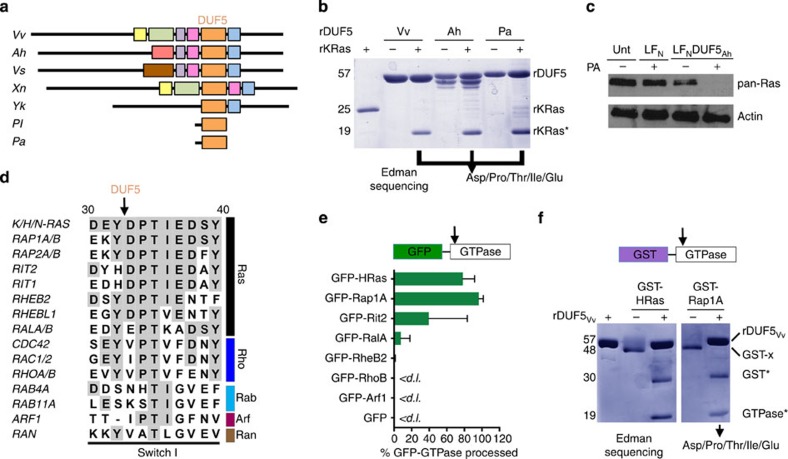
DUF5 homologues and other GTPase substrates. (**a**) Schematic diagram of DUF5 (orange) within the mosaic architecture of effector domains in MARTX toxins from *V. vulnificus (Vv), A. hydrophila (Ah), Vibrio splendidus (Vs), Xenorhabdus nematophila (Xn)* and *Yersinia kristensii (Yk)* or as stand-alone proteins in *Photorhabdus luminescens* (Pl) and *P. asymbiotica* (Pa) as previously described^17^,^20^. (**b**) *In-vitro* cleavage of 10 μM KRas with 10 μM rDUF5 from various species. (**c**) LF_N_DUF5_Ah_ tested for *in-vivo* loss of all Ras isoforms after 24 h under the same conditions as in Fig. 1 **b**. (**d**) Amino acid identity in Switch I regions of representative GTPases (left) from five major Ras families (right). (**e)** Bar graph of per cent GFP-fusion protein cleaved after delivery of LF_N_DUF5_Vv_+PA, quantified from immunoblots (Supplementary Fig. 5). Error bars indicate mean±s.d. (*n*=3). (**f**) Representative *in-vitro* cleavage (*n*=3) of GST-fusion proteins to release GST*. Negative cleavage reactions for nine other substrates are shown in Supplementary Fig. 6.

Domains similar to DUF5_Vv_ have been identified in other bacterial species (Fig. 3a). To determine whether Ras processing is a conserved function among bacteria, the effector domain from the *Aeromonas hydrophila* MARTX toxin (rDUF5_Ah_) and a hypothetical effector protein from insect pathogen *Photorhabdus asymbiotica* (rDUF5_Pa_) were also purified and tested for proteolytic activity. Both proteins were found to cleave rKRas *in-vitro* with cleavage occurring between Y32 and D33 (Fig. 3b). As further validation, DUF5_Ah_ was fused to LF_N_ (LF_N_DUF5_Ah_). This protein induced both cytotoxicity and Ras cleavage in intoxicated cells when delivered to cells by PA (Fig. 3c). Thus, DUF5 represents a new family of bacterial toxin effectors that catalyses site-specific processing of the Switch I region of all three major isoforms of Ras independently of any other cellular proteins.

### Rap1 is also a substrate for DUF5_Vv_

Other bacterial protein toxins are known to promiscuously target a wide range of small GTPases and other cellular proteins^15^. As the amino acid sequence of the Switch I region of Ras is well conserved across Ras subfamily members (Fig. 3d), it was considered that DUF5_Vv_ might also cleave other small GTPases. To test this, representative Ras subfamily small GTPases fused via their N termini to enhanced GFP (EGFP) were ectopically expressed in HEK 293T cells and anti-GFP antibody was used to detect the released N-terminal fragment. In cells treated with LF_N_DUF5_Vv_+PA, EGFP-HRas and EGFP-Rap1 were both cleaved with >80% efficiency. Processing of another Ras subfamily member, Rit2, was also detected in this assay, but with inconsistent efficiency, resulting in a large s.d. across multiple experiments (Fig. 3e). This indicates that Rit2 may be a low-affinity substrate resulting in experimental variation dependent on the ratio of toxin to GFP-Rit2 in each cell or sample (Fig. 3e). Other small EGFP-GTPases (RalA, RheB2, RhoB and Arf1) showed no cleavage, indicating they are not *in-vivo* substrates (Fig. 3e and Supplementary Fig. 5).

DUF5_Vv_ specificity for Ras and Rap1 was further verified biochemically. Small Ras GTPases covering the diversity of Ras subfamilies were purified as substrates for *in-vitro* assay to assess whether rDUF5_Vv_ could catalyse their cleavage. Among the 11 GTPases tested (Supplementary Fig. 6), only Rap1 was confirmed as a DUF5_Vv_ substrate, with cleavage occurring after Y32 (Fig. 3f), whereas Rit2 was not cleaved at all, confirming that in cells this is a low-affinity substrate (Supplementary Fig. 6). Other GTPases belonging to the Ras, Rho, Rab and Ran subfamilies were not processed (Supplementary Fig. 6). Thus, DUF5_Vv_ is a specific protease that preferably cleaves Ras and Rap1 without cellular co-factors. The detection of Rap1 as an additional substrate is especially interesting for bacterial pathogenesis, as Rap1 activates ERK in response to bacterial components other than lipopolysaccharide and is critical for macrophage phagocytosis^31^,^32^.

### DUF5_Vv_ targets Ras during bacterial infection

**Figure 4:**
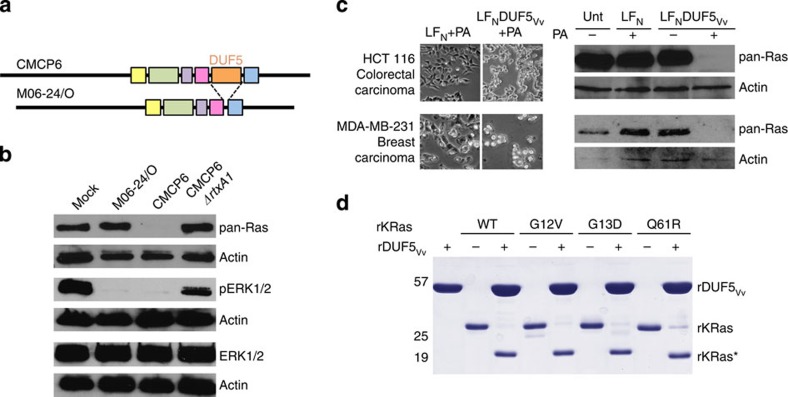
DUF5_Vv_ during bacterial infection and as a potential treatment of malignancies. **(a**) MARTX toxin effector domain configuration in *V. vulnificus* isolates CMCP6 (DUF5_Vv_^+^) and M06-24/O (DUF5_Vv_^−^). (**b**) Representative immunoblots (*n*=2) of lysates from cells incubated with *V. vulnificus* as indicated and probed for Ras cleavage and ERK1/2 dephosphorylation. (**c**) Phase-contrast images and immunoblot detection of Ras from HCT116 and MDA-MB-231 cells treated as indicated for 24 h. (**d**) *In-vitro* processing of 10 μM rKRas with mutations as indicated.

Given the importance of Ras and Rap1 in the host response to bacterial infection, it is not surprising that DUF5_Vv_ was previously shown to contribute to *V. vulnificus* virulence^19^. The strain CMCP6 produces a MARTX toxin that carries five effector domains, including DUF5_Vv_ in the fifth position. By contrast, M06-24/O produces a toxin with only four effector domains (Fig. 4a), having undergone a genetic recombination that resulted in an in-frame deletion of the DNA sequence for the DUF5_Vv_ domain^19^,^33^. As a result of the loss of DUF5_Vv_, M06-24/O is tenfold less virulent than CMCP6 (ref. 19). The increased virulence of CMCP6 was found to be specifically due to DUF_Vv_^19^, even though both toxin forms induce cellular necrosis^34^,^35^ (Supplementary Fig. 7).

To link this defect in virulence to Ras activation and demonstrate that Ras can be processed during normal toxin delivery, HeLa cells were co-cultured for 1 h with *V. vulnificus* and proteins in cell lysates were analysed by western blotting. Cells treated with wild-type bacteria producing full-length active MARTX toxin no longer showed detectable Ras or pERK1/2. This inactivation was dependent on an intact *rtxA1* toxin gene, as a null mutation in *rtxA1* of *V. vulnificus* CMCP6 did not show loss of detectable Ras or pERK1/2. Further, co-culture of cells with *V. vulnificus* M06-24/O, which produces the MARTX toxin naturally missing DUF5_Vv_, did not affect Ras, linking this MARTX-dependent activity specifically to the DUF5_Vv_ effector domain. Interestingly, cells treated with M06-24/O unexpectedly still showed a reduction of pERK1/2, revealing that these multifunctional toxins probably have redundant strategies to inactivate ERK during infection (Fig. 4b).

### Oncogenic KRas is processed by DUF5_Vv_

Point mutations resulting in constitutive activation of Ras have long been associated with many different types of adenocarcinomas^5^,^6^,^7^. The discovery of a novel bacterial toxin mechanism to halt cell proliferation through processing of Ras is not only important for understanding the function of bacterial toxins during infection but also presents an opportunity to potentially target Ras during carcinogenesis through delivery of DUF5. This strategy would be most successful if mutant forms of Ras found in cancer cells are also DUF5 substrates.

When HCT116 colorectal carcinoma cells, which express KRas with a G13D mutation, were intoxicated with PA in combination with LF_N_DUF5_Vv_ (Fig. 4c) or LF_N_DUF5_Ah_ (Supplementary Fig. 8), significant cell morphological changes were observed and Ras was undetectable by western blotting. Similar results were obtained with the breast cancer cell line MDA-MB-231 that likewise carries the KRas G13D mutation. This cell line also contains a G464V mutation in B-Raf^36^, an effector of both Ras and Rap1 (ref. 37), demonstrating that DUF5_Vv_ can effectively intoxicate cells even if they have additional activating mutations downstream of Ras and Rap1.

As further demonstration that DUF5_Vv_ could be employed as a cancer treatment, rKRas was modified to carry three of the most common Ras mutations associated with tumorigenesis: G12V, G13D or Q61R^7^. All three mutant forms of KRas were confirmed as *in vitro* substrates for rDUF5_Vv_-dependent site-specific processing (Fig. 4d). Thus, the ability of DUF5_Vv_ to cleave KRas is unaffected by the most common *RAS* mutations. Overall, these data show that cells carrying constitutively active forms of Ras are not protected from DUF5_Vv_ cytotoxicity and thus DUF5_Vv_ is a valid candidate for use as an anti-tumour agent.

## Discussion

MARTX toxins are large bacterial toxins that carry multiple effector domains, each with a specific enzymatic activity. DUF5_Vv_, the extra effector domain of the MARTX toxin from the most virulent strains of the sepsis-causing pathogen *V. vulnificus*, was previously shown to be highly cytotoxic for mammalian cells, although the mechanism of this cytotoxicity was unknown^20^. In this work, we demonstrate that DUF5_Vv_ is a representative member of a new family of bacterial toxin effectors that catalyse site-specific processing of the Switch I region of Ras and Rap1. Activated Ras or Rap1 would normally interact with downstream effectors such as c-Raf, to stimulate the phosphorylation of ERK1/2. In particular, Y32 in the Switch I region plays an important role in stabilizing the GTP-bound form of Ras and its interaction with the Raf kinases^27^. Thus, it is predicted that DUF5_Vv_ cleavage between Y32 and D33 would destabilize the Switch I and presumably the interactions of Ras and Rap1 with their binding partners. As Ras and Rap1 form parallel pathways that relay signals from surface receptors and guanine nucleotide exchange factors to activate ERK1/2, disabling both small GTPases simultaneously nullifies all downstream signalling pathways^38^, resulting in the complete loss of pERK1/2 in DUF5_Vv_-treated cells. In the context of bacterial infection, this is important to inactivate innate immune responses, accounting for the direct linkage of this toxin effector domain to virulence of *V. vulnificus*. We propose that the DUF5 effector domain be renamed RRSP for Ras/Rap1-specific protease, acknowledging its site-specific processing of the Switch I region of Ras and Rap1.

As small GTPases are responsible for regulating essential cell functions, many other bacterial protein toxins and effectors target GTPases by posttranslational modification or by manipulating their function^15^. However, few of these toxins target Ras specifically, for example, *Pseudomonas aeruginosa* ExoS ADP-ribosylates R41 of Ras and Rap^39^,^40^,^41^, and thereby directly inhibits phagocytosis in mice^42^. However, ExoS also has broad substrate recognition including other GTPases^43^ and other proteins such as moesin and vimentin^16^,^44^,^45^. Similarly, *Clostridium sordellii* lethal toxin TcsL (also known as LT) has been shown to glucosylate Ras at T35 in the Switch I^46^,^47^ resulting is cellular apoptosis^48^. In addition, TcsL UDP-glucosylates other small Ras, Rap, Ral, Rho and Rac GTPases with some specificity differences depending on strain^49^. Through a similar process, *Clostridium perfringens* large toxin TpeL modifies T35 of Ras and, to a lesser extent, Rap1 and possibly Rac1, except it preferentially uses UDP-*N*-acetylglucosamine as a sugar donor^50^,^51^.

The unique feature of RRSP demonstrated here is its irreversible mechanism of action by cleaving rather than modifying Ras and Rap1. The biochemical basis for the specificity of RRSP for Ras and Rap1 should be explored further in the future. Although it is possible that the specificity is dictated by the conservation of the amino acid sequence in the Ras and Rap1 Switch I regions, it is more likely to be that recognition of the target is multifactorial depending on a multifaceted protein–protein interaction between RRSP and Ras or Rap1. This possibility is supported by studies of *Clostridium difficile* toxin TcdB recognition of RhoA as a substrate for glucosylation, which is mediated in part by specificity for target residue T37 in the Switch I region^52^, but also by Ser73 outside the Switch I^53^. In addition, amino acids of TcdB essential to discriminate substrate are found outside the catalytic site, further indicating that specificity of TcdB from Rho in not driven solely by the Switch I sequence^54^.

In addition to protein–protein interactions, specificity of RRSP for Ras and Rap1 may include spatial localization to anionic membranes or specificity for the active or inactive state conformation when bound to GTP or GDP, respectively. However, in cells, we routinely observed 100% processing of all Ras isoforms in as little as 30 min and we also observed 100% cleavage of KRas G12V, G13D and Q61R *in-vitro*, despite not controlling the GTP or GDP state using buffers. These data would seem to support the hypothesis that RRSP can target both active and inactive forms of Ras and thereby access both membrane and cytoplasmic pools of Ras. In addition, as the Switch I region undergoes structural changes with activation state, and both active and inactive forms of Ras seem to be substrates for RRSP, we suppose specificity is at least in part driven by protein–protein interaction outside the Switch I region and this will be explored in the future through detailed structural and binding studies.

A critical question for bacterial infection is how the processing of Ras and Rap1 contributes to increased virulence. The MARTX toxin of *V. vulnificus* is known to play a role during infection both in paralysing phagocytic cells^55^ and in breaching the epithelial barrier to promote spread of the bacterium from the intestine to other organs^56^,^57^,^58^. Overall, small GTPases play a central role in the barrier function of epithelial layers such that loss of this control could contribute to bacterial spread across the intestinal barrier^15^. In particular, Ras and Rap1 are essential for sensing and signalling pathogen-associated molecular patterns and for regulating inflammatory responses of the host organism^15^,^59^. In addition, Ras and Rap1 function to activate the ERK1/2 pathway in response to bacterial components such as LPS to stimulate macrophage phagocytosis^31^,^32^. In the context of bacterial infection, inhibition of these cascades would slow down the host response, such that *V. vulnificus* strain that carry this domain are more virulent^19^.

A final impact of our discovery is the possibility that the RRSP effector domain could be deployed across the cell membrane to specifically target tumour cells using different delivery strategies. More than three decades after the discovery of Ras implication in cancer development, targeting Ras remains one of the hardest challenges of cancer research and drug discovery^7^. Here, we propose that proteins in this new RRSP effector family could be employed immediately as research tools, but in the future developed as new anti-cancer therapeutic agents. Of particular immediate interest, re-engineered PA selectively targeting cancer cells could be used to deliver LF_N_DUF5 into cells to destroy Ras and thereby deregulate tumour growth and proliferation. This approach has already been validated in cell systems in which PA was fused to the epidermal growth factor for delivery of LF_N_-tethered cargo into cancer cells with upregulated expression of the epidermal growth factor receptor^60^. This system has also been proven with PA modified to bind to the HER2 receptor, a protein strongly upregulated in tumour cells, in particular breast cancers^61^. As alternative future approaches, RRSP effector domains could be fused to specific antibodies for use as an immunotoxin^62^, or expressed and delivered by *Salmonella* bacteria that home to solid tumours^63^. It could also be expressed by viruses engineered to specifically infect cancer cells^64^. The ability of RRSP to cleave both normal and mutant forms of Ras indicates that any developed reagent could be successful whether used for Ras cancers, non-Ras cancers, or other Ras-associated diseases.

## Methods

### General molecular biology techniques

*E. coli* DH5α and TOP10 cells (Life Technologies) were grown at 37 °C in Luria–Bertani liquid or on agar medium supplemented with either 100 μg ml^−1^ ampicillin or 50 μg ml^−1^ kanamycin, as needed. Common reagents were obtained from Sigma-Aldrich, Fisher or VWR, and common restriction enzymes and polymerases were obtained from New England Biolabs or Life Technologies. Custom DNA oligonucleotides were purchased from Integrated DNA Technologies (Coralville, IA). Plasmids were prepared by alkaline lysis followed by precipitation in ethanol or purified using Epoch spin columns according to the manufacturer's recommended protocol. A Qiagen Midi Prep kit was used for preparation of plasmids used in yeast transformations. Plasmids were introduced into *E. coli* by electroporation and into HeLa cells by transfection using polyethylenimine (PEI).

### Yeast non-essential gene deletion screen

The Life Technologies YKO yeast deletion library covering all non-essential genes was replicated from stocks at the Northwestern University High Throughput Analysis Laboratory using a Genetix QPixII Automatic colony picker. Each strain from the library was subsequently grown in 1 ml yeast extract peptone dextrose with addition of 50 μg ml^−1^ G418. After overnight growth at 30 °C with agitation, each strain was transformed with plasmid pYC-C2 using a PLATE solution method and transformants were selected on synthetic complete agar without uracil and with 2% glucose to repress DUF5_Vv_-C2 expression. Colonies were patched with toothpicks onto synthetic complete agar supplemented with 2% galactose and 1% raffinose to induce DUF5_Vv_-C2 expression. Initial positive selection was defined as yeast that formed a patch when grown on galactose. These were subsequently rescreened in a dilution plating assay as previously described^20^ and those with a plating efficiency comparable to a strain transformed with empty vector were considered validated hits. Identified strains were analysed and classified based on information in the *Saccharomyces* Genome Database (www.yeastgenome.org), last accessed on 25 October 2014.

### Intoxication of cells with proteins fused to LF_N_

HeLa, HCT116 and HEK293 cells were grown at 37 °C with 5% CO_2_ in DMEM medium (Life Technologies) with 10% fetal bovine serum (Gemini Bio-Products, West Sacramento, CA), 100 U ml^−1^ penicillin and 1 μg ml^−1^ streptomycin. Purification of LF_N_, LF_N_DUF5_Vv_ and LF_N_DUF5_Ah_ has been previously described^20^. PA purified as previously described^65^ was provided by Shivani Agarwal (Northwestern University). Cell lines were seeded overnight into tissue culture-treated dishes and flasks, except for HCT116 cells, which were seeded for 48 h. Before intoxication, the media was exchanged for fresh media and then 7 nM PA and 3 nM LF_N_-tagged toxins were added to the media and incubated for the times indicated in the legend at 37 °C with 5% CO_2_. Cells were imaged at × 10 at times indicated in the legend using a Nikon TS Eclipse 100 microscope equipped with a Nikon CoolPix 995 digital camera or processed for western blotting or colony formation as detailed below.

### Western blotting

A total of 2.5–5 × 10^4^ treated cells were washed with PBS, then resuspended in 120 μl of 2 × Laemmli sample buffer and boiled for 10 min. Ten microlitres of lysate were separated by SDS–PAGE and transferred to nitrocellulose (Amersham) using the Bio Rad Trans-Blot Turbo system. Nitrocellulose membranes were blocked overnight at 4 °C in 5% (w/v) powdered milk diluted in Tris-buffered saline containing 0.001% Tween-20 (TBS-T). Immunodetection of proteins was conducted as previously described^20^, using primary antibodies purchased from Cell Signaling Technologies (p44/42 MAPK (ERK1/2) rabbit mAb 137F5 (1:1,000), phospho-p44/42 (ERK1/2) rabbit mAb 197G2 (1:1,000), p38 MAPK rabbit polyclonal 9212 (1:1,000) and phospho-p38 rabbit mAb 12F8 (1:1,000)), EMD Millipore (pan-Ras mouse mAb RAS10 (05-516, 1:1,000)), Thermo Scientific (HRas PA5-22392 (1:1,000), KRas PA5-27234 (1:1,000) and NRas PA5-28861 (1:1,000)) and Sigma-Aldrich (H6908 rabbit polyclonal (1:5,000), actin mouse mAb AC-40 (1:1,000) and Tubulin T6074, (1:10,000)). Antibody binding to proteins was detected using anti-mouse (1:5,000) or anti-rabbit (1:5,000) secondary antibodies conjugated to horseradish peroxidase from Jackson Immuno Research and developed using SuperSignal WestPico chemiluminescent reagents (Thermo Scientific) and X-ray film. For serial detection of proteins and detection of the actin-loading controls from the same nitrocellulose membrane, membranes were washed in TBS-T for 10 min and then stripped of antibody by washing the membrane for 10 min with stripping buffer (1.5% glycine, 1% Tween-20, 0.1% SDS). After two more 10-min washes with TBS-T, the membrane was re-probed for other proteins. Tubulin-loading controls were performed by cutting the membrane horizontally to separate the upper loading control portion containing tubulin from the lower portion containing the small Ras family GTPases. Uncropped western blottings are shown in Supplementary Figs 9–17.

### Ras G-LISA

Active (GTP-bound) Ras in intoxicated cells was measured using the Ras G-LISA activation colorimetric assay kit from Cytoskeleton, Inc. (Denver, CO). HeLa cells were seeded into 10-cm^2^ tissue culture-treated dishes and grown to ∼80% confluency, at which time the cells were intoxicated with LF_N_ proteins in combination with PA for 24 h as described above. Cells were collected in the lysis buffer and total protein content was determined by the Precision Red assay using reagents supplied with the kit. The lysate was frozen in a dry ice-ethanol bath and stored at −80 °C. Active Ras in each lysate was then determined according the manufacturer's protocol. This kit used the pan-Ras RAS10 mAb for detection of active Ras and this antibody was subsequently obtained directly from Millipore for western blotting detection of Ras as described above.

### Clonogenic colony-formation assay

A total of 10^5^ HeLa cells were seeded into six-well dishes overnight, intoxicated with LF_N_ protein as described above and assessed by a clonogenic colony-formation assay as described previously^66^. Briefly, cells were released from wells with 0.25% trypsin/EDTA (Sigma), counted in a hemocytometer and then diluted. The number of cells indicated was replated in fresh media in duplicate. After 14 days, cells were fixed with 70% ethanol and stained with 0.5% crystal violet, and colonies of more than 50 cells were counted. The surviving fraction was compared with cells treated with LF_N_+PA.

### Ectopic expression of HA-tagged Ras isoforms

Plasmids for ectopic expression of HA-HRas (pcDNA3-HA-HRas_wt, 14723) and HA-NRas (pCGN NRas wt, 39503) were obtained from Addgene (Cambridge, MA). Plasmids for overexpression of HA-KRas and HA-KRas G12V were obtained from Athanasios Vassilopoulos (Northwestern University). Plasmid DNA (2 μg) was mixed with 90 μl PEI diluted in incomplete DMEM media, vortexed 15 times and then incubated for 15 min at room temperature. Seven hundred microlitres of complete DMEM were added into the plasmid–PEI mix and the whole volume was added to HeLa cells. After 24 h, cells were intoxicated as described above.

### Immunoprecipitation of HA-HRas and mass spectrometry

HeLa cells, either untreated or intoxicated with LF_N_DUF5_Vv_+PA as described above, were washed with cold PBS and then resuspended in RIPA buffer (50 mM Tris-HCl pH 7.5, 150 mM NaCl, 0.1% SDS, 0.5% sodium deoxycholate, 1% Triton and ‘cOmplete' protease inhibitors). HeLa cell lysates were incubated with 50 μl of anti-HA agarose beads (Sigma) for 2 h at 4 °C under mild agitation. Beads were then washed five times with 500 μl of RIPA buffer and five times with 500 μl of washing buffer (50 mM Tris-HCl pH 7.5, 150 mM NaCl). Proteins bound to the beads were eluted with 3 M sodium thiocyanate buffer (50 mM Tris-HCl pH 7.5, 150 mM NaCl). Elution fractions were analysed by SDS–PAGE followed by Coomassie staining or immunoblotting using anti-HA and isotype-specific anti-HRas antibody as described above. The smaller HRas band was excised from the gel, put in water and then frozen for shipping. Trypsin digestion followed by liquid chromatography–tandem mass spectrometry on the Thermo LTQ-FT Ultra spectrophotometer was conducted at the University of Illinois at Chicago Mass Spectrometry, Metabolomics and Proteomics Facility according to their standard protocols.

### Preparation of 6xHis- or GST-tagged small GTPases

DNA sequences corresponding to *KRas* (*KRas4B*, *NP_004976.2*), *HRas* (*NP_001123914.1*) and *NRas* (*NP_002515.1*) genes were amplified from templates as described above, using primers designed for ligation-independent cloning, and the products were cloned into the pMCSG7 expression vector by ligation-independent cloning^67^. The G12V, G13D and Q61R mutations were introduced by site-directed mutagenesis using the pMCSG7-KRas vector as a template. Primers are listed in Supplementary Table 1. Plasmids were confirmed to be accurate by DNA sequencing and then transformed into *E. coli* BL21(DE3).

Cultures of *E. coli* were grown at 25 °C in Terrific Broth supplemented with 100 μg ml^−1^ ampicillin to an OD_600_ of 0.6–0.7 and then induced with 1 mM isopropyl-β-D-thiogalactoside and growth was continued at 18 °C for ∼18 h. Bacteria were harvested by centrifugation, re-suspended in buffer A1 (50 mM Tris pH 7.5, 500 mM NaCl, 10 mM MgCl_2_, 0.1% Triton X-100, 5 mM β-mercaptoethanol) and lysed by sonication. After centrifugation at 30,000*g* for 30 min, the soluble lysate was loaded onto a 5-ml HisTrap column using the ÄKTA protein purification system (GE Healthcare). The column was washed with buffer B1 (10 mM Tris pH 7.5, 500 mM NaCl, 10 mM MgCl_2_, 50 mM imidazole) followed by elution in the same buffer with 500 mM imidazole (buffer C1). Proteins were further purified by size-exclusion chromatography (Superdex 200 (26/60), GE Healthcare) in buffer D1 (10 mM Tris-HCl pH 7.5, 500 mM NaCl, 10 mM MgCl_2_, 5 mM β-mercaptoethanol).

GST-fusion GTPases were obtained from Seema Mattoo (Purdue University, IN), and expressed and purified as previously reported^68^.

### Preparation of 6 × His-tagged DUF5 proteins

DNA sequences corresponding to DUF5_Vv_ (*V. vulnificus* CMCP6—MARTX_Vv_ Q3596-L4089, NP_759056.1), DUF5_Ah_ (*A. hydrophila* ATCC7966—MARTX_Ah_ P3069-V3570—locus WP_011705266) and DUF5_Pa_ (*P. asymbiotica* ATCC43949—P41-V532 locus WP_011705266) were amplified from their respective genomes using primers designed for ligation-independent cloning and the products were cloned into the pMCSG7 expression vector by ligation-independent cloning^67^.

Primers are listed in Supplementary Table 1. Plasmids were confirmed to be accurate by DNA sequencing and then transformed into *E. coli* BL21(DE3). Cultures were grown in Terrific Broth supplemented with 100 mg ml^−1^ampicillin at 37 °C until OD_600_=0.7–0.8 and then induced with 1 mM isopropyl-β-D-thiogalactoside at 18 °C for ∼18 h. Proteins were purified as described above for Ras proteins, except all buffers were adjusted to pH 8.3 instead of 7.5.

### *In-vitro* cleavage assay and N-terminal sequencing

rKRas, rHRas, rNRas and GST-fused small GTPases were incubated with rDUF5 proteins at equimolar concentrations (10 μM) in 10 mM Tris pH 7.5, 500 mM NaCl, 10 mM MgCl_2_ at 37 °C for 10 min, unless otherwise indicated. Reactions were stopped by adding 6 × Laemmli sample buffer and incubating the sample at 90 °C for 5 min. Proteins were separated on 18% SDS–polyacrylamide gels and visualized using Coomassie stain. Cleavage of Ras isoforms and GTPases was quantified from scanned gels using NIH ImageJ 1.64. To identify the cleavage site, proteins separated by 18% SDS–polyacrylamide were transferred onto a polyvinylidene difluoride membrane. After Coomassie staining, processed bands were excised from the membrane and sequenced on an ABI 494 Procise Protein Sequencer (Applied Biosystem) using automated Edman degradation at the Tufts University Core Facility.

### *In-vivo* cleavage assay of small GTPases

DNA sequences coding for HRas, Rap1A, Rit2, RalA, Rheb2A, RhoB and Arf1 were amplified from plasmids for overexpression of GST-GTPases as described above^68^. Products were inserted into pEGFP-C3 (Clontech) using SmaI and the Gibson Assembly Cloning Kit (NEB). HEK 293T cells were transfected with the resulting plasmids as described above. After 24 h, cells were intoxicated with LF_N_ proteins and cleavage detected using monoclonal GFP-HRP antibody (Miltenyi Biotec) as described above. The amount of cleaved protein as a per cent of total GFP protein was quantified from scanned gels using NIH ImageJ 1.64 and data were normalized to the pixels detected in the absence of intoxication.

### Bacterial challenge of HeLa cells

*V. vulnificus* rifampicin-resistant isolates of strains CMCP6, M06-24/O and CMCP6Δ*rtxA1* (ref. 19) were grown at 30 °C in Luria–Bertani medium with 50 μg ml^−1^ rifampicin. Overnight cultures were diluted 1:500 and grown at 30 °C with shaking until the OD_600_ reached 0.55–0.6. Bacteria from 1 ml were pelleted at 1,800*g* for 4 min, washed once in PBS and then resuspended in 1 ml PBS. Media were exchanged over 5 × 10^4^ HeLa cells previously seeded in 12-well plates overnight for antibiotic-free media. *V. vulnificus* in PBS (multiplicity of infection=100) or an equal volume of buffer was added to media over cells and plates were centrifuged at 25 °C for 5 min at 500*g*. After 60 min, cells were photographed as described above, to assess rounding before collection of lysate and western blotting of proteins in 15 μl of lysate as described above.

In a separate set of experiments, cells in phenol red-free DMEM with 10% fetal bovine serum but no antibiotics were incubated up to 4 h. At 1-h intervals, 50 μl of supernatant were sampled and assayed for release of lactate dehydrogenase using the Cytotox 96 Non-Radioactive Cytotoxicity Assay (Promega), according to the manufacturer's protocol. Per cent cell lysis was calculated as the lactate dehydrogenase release in the sample divided by a positive control lysed with 0.1% Triton X-100.

## Additional information

**How to cite this article:** Antic, I. *et al*. Site-specific processing of Ras and Rap1 Switch I by a MARTX toxin effector domain. *Nat. Commun.* 6:7396 doi: 10.1038/ncomms8396 (2015).

## Supplementary Material

Supplementary Information Supplementary Figures 1-17 and Supplementary Table 1
