# Supplementary material for: Site-specific processing of Ras and Rap1 Switch I by a MARTX toxin effector domain
Source: Nat Commun. 2015 Jun 8;6:7396. doi: 10.1038/ncomms8396 (PMC4468845; doi:10.1038/ncomms8396)
Supplement: Supplementary Information — Supplementary Figures 1-17 and Supplementary Table 1 [file ncomms8396-s1.pdf]

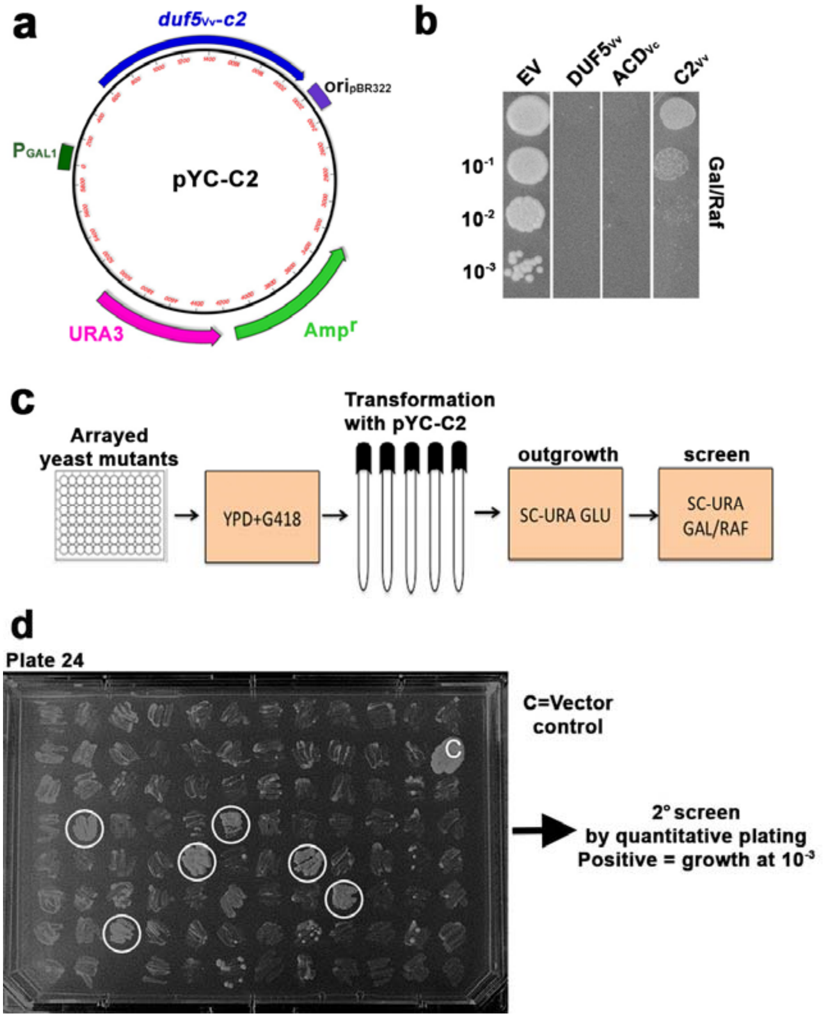

Supplementary Figure 1 | Schematic summary of yeast deletion screen.

(**a**) Diagram of pYC-C2 plasmid expressing $DUF5_{Vv}$-C2 under control of the GAL1 galactose-inducible promoter. (**b**) Plating efficiency of *S. cerevisiae* InvSc2 expressing $DUF5_{Vv}$-C2 ($C2_{Vv}$) compared to yeast transformed with empty vector (EV) and the more toxic full-length $DUF5_{Vv}$ and actin crosslinking domain from *V. cholerae* (ACD_{Vc}), which eliminates the actin cytoskeleton (Geissler B, et al. *Mol Microbiol* **73**, 858-868 (2009)). (**c**) Schematic showing the arrayed library of non-essential deletion strains transformed with pYC-C2, followed by selection on glucose to repress expression of $DUF5_{Vv}$-C2. The resulting yeast colonies were patched onto galactose and raffinose to induce expression. (**d**) Plate 24 of the library, showing the initial screen yeast transformed with empty vector (C) and strains selected for secondary screening by quantitative plating (circled).

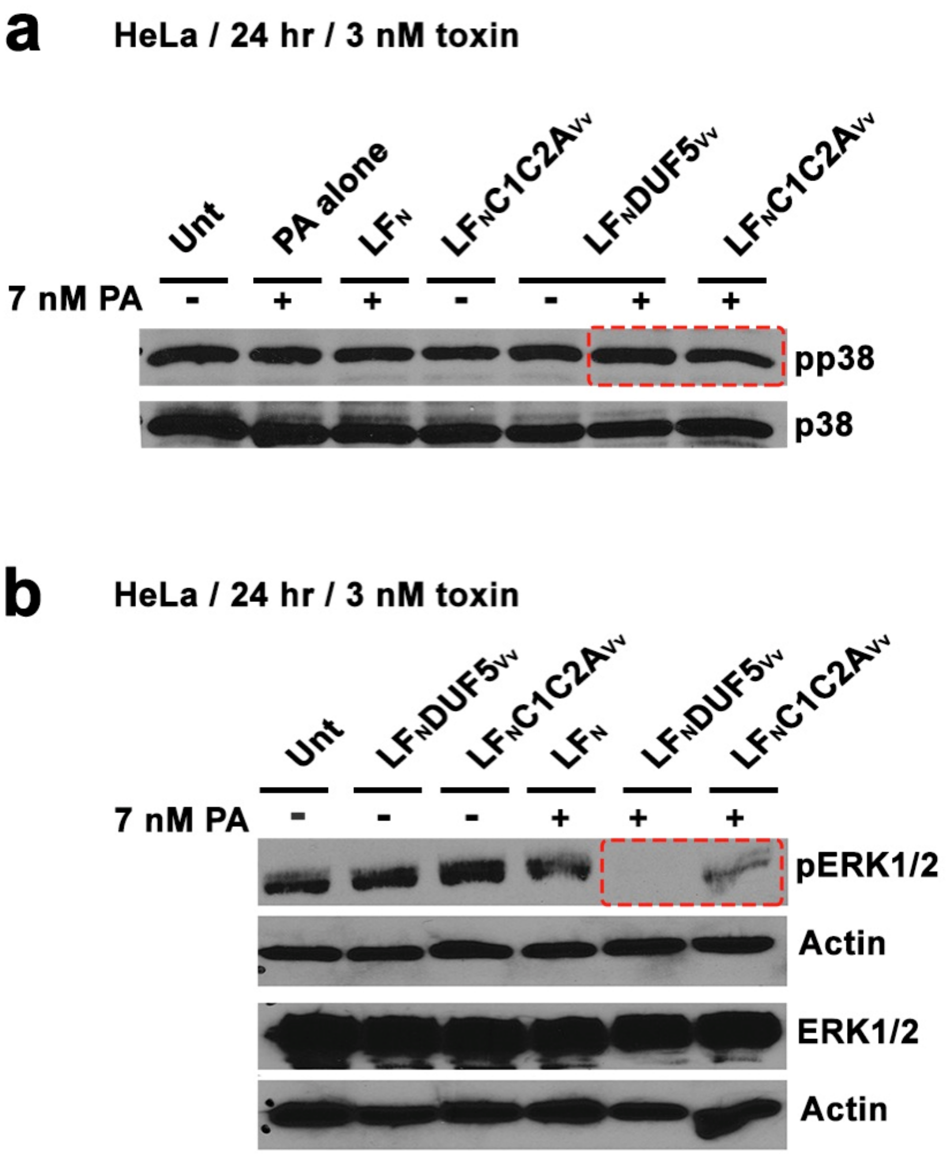

Supplementary Figure 2 │ DUF5$_{Vv}$ inhibits ERK1/2 phosphorylation, but not p38.

(**a**,**b**) Representative immunoblots (*n*=2) of lysates from cells treated as indicated for 24 h. Red boxes highlight differences in phospho-p38 (pp38) and phospho-ERK1/2 (pERK1/2) levels. Note that Panel **b** is the same figure from which lanes were removed to align with other western blots in Fig. 1b.

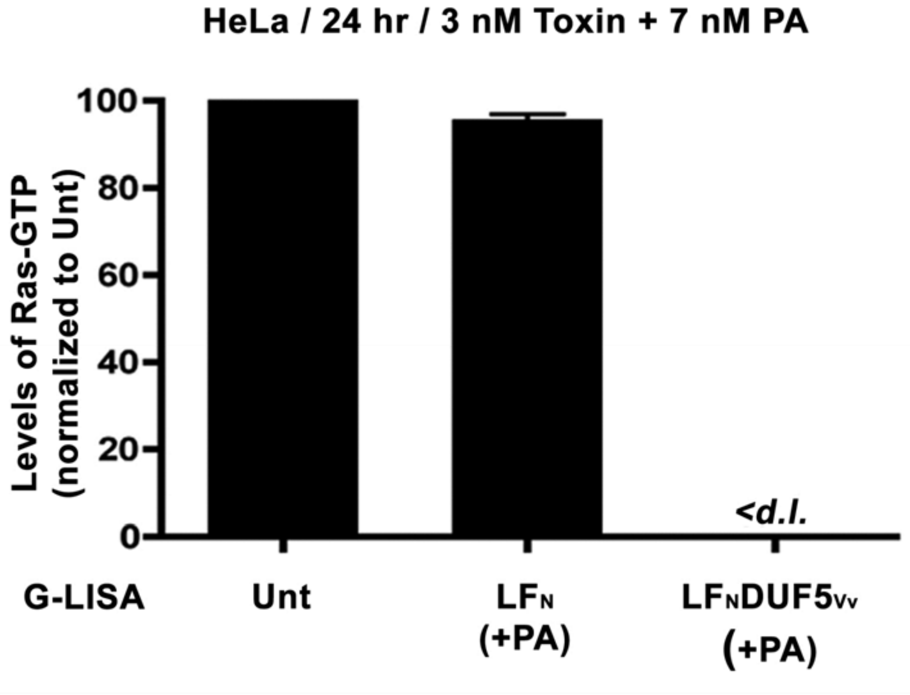

Supplementary Figure 3 │ HeLa cells treated with DUF5$_{Vv}$ lack active (GTP-bound) Ras. Bar graph of relative detection of active GTP-bound Ras (all isoforms) by G-LISA. Failure to detect active Ras was ultimately explained by the complete absence of Ras detectable by the monoclonal RAS10 antibody provided with the assay kit.

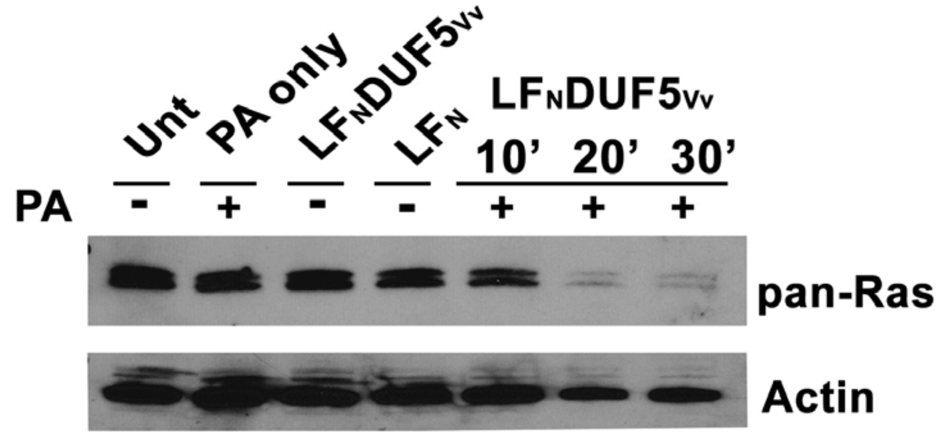

Supplementary Figure 4 │ Ras inactivation by DUF5$_{Vv}$ occurs rapidly.

Immunoblot of lysates from cells treated for time indicated. Control samples (first four lanes) were collected 30 minutes after intoxication.

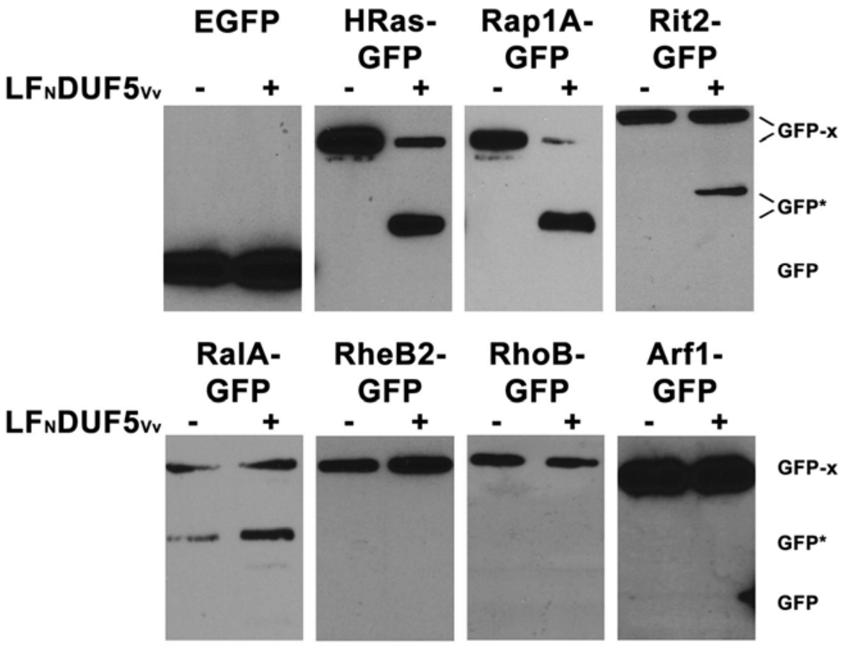

Raw data used for bar graph of EGFP-GTPase from Fig. 3e

	EGFP		HRas		Rap1A		Rit2	
**$LF_N DUF5_{Vv}$**	-	+	-	+	-	+	-	+
	0	0	0	69.3	0	90.3	0	31.8
	0	0	23.6	95.2	0	100	0	86.7
	0	0	0	93.7	0	98.7	0	0
	RalA		RheB2A		RhoB		Arf1	
**$LF_N DUF5_{Vv}$**	-	+	-	+	-	+	-	+
	33.6	52.3	10.4	12.1	0	0	0	0
	26	24.2	0	0	0	0	0	0
	0	6.1	0	0	0	0	0	0

Supplementary Figure 5 │$DUF5_{Vv}$ specificity against GFP-tagged small GTPases.

HEK 293T cells transfected to express small GTPases with N-terminal EGFP-fusion as indicated were either untreated (-) or intoxicated with $LF_N DUF5_{Vv}$ in combination with PA (+) for 24 h, at which time cell lysates were probed with anti-EGFP antibody. For triplicate blots, GFP* and GFP-x bands were quantified by Image J 1.64 and percent cleavage determined as GFP*/(GFP*+GFP-x). For Fig. 3e, samples were normalized to untreated cells to account for closely sized non-specific bands or natural breakdown. Raw pixel data is shown in table.

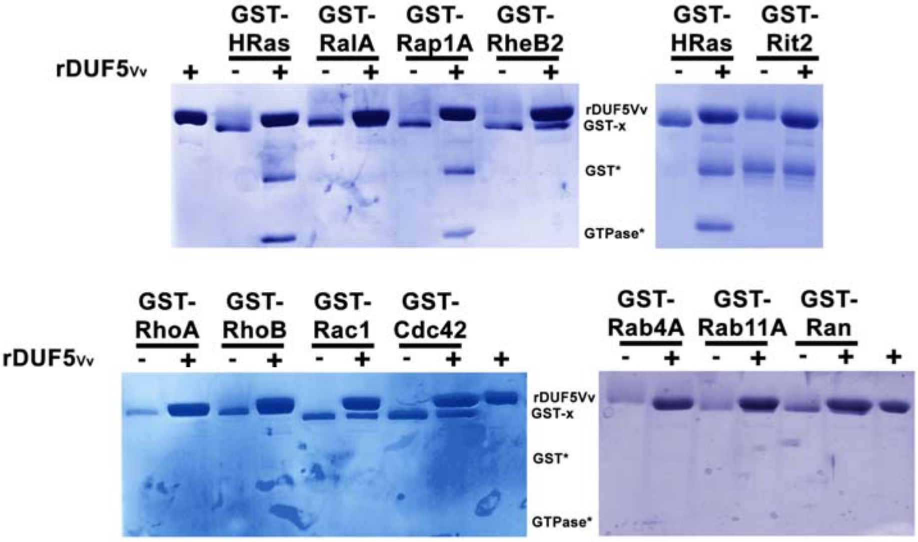

Supplementary Figure 6 │ DUF5$_{Vv}$ specificity against GST-tagged small GTPases.

In vitro processing of 10 μM purified small GTPases with N-terminal fusion of GST (GST-x) to two fragments (GST* and GTPase*) by 10μM rDUF5$_{Vv}$ for 10 min. This extended figure shows representative data (*n*=3). Only the positive samples, HRas and Rap1A, are duplicated in Fig. 3f.

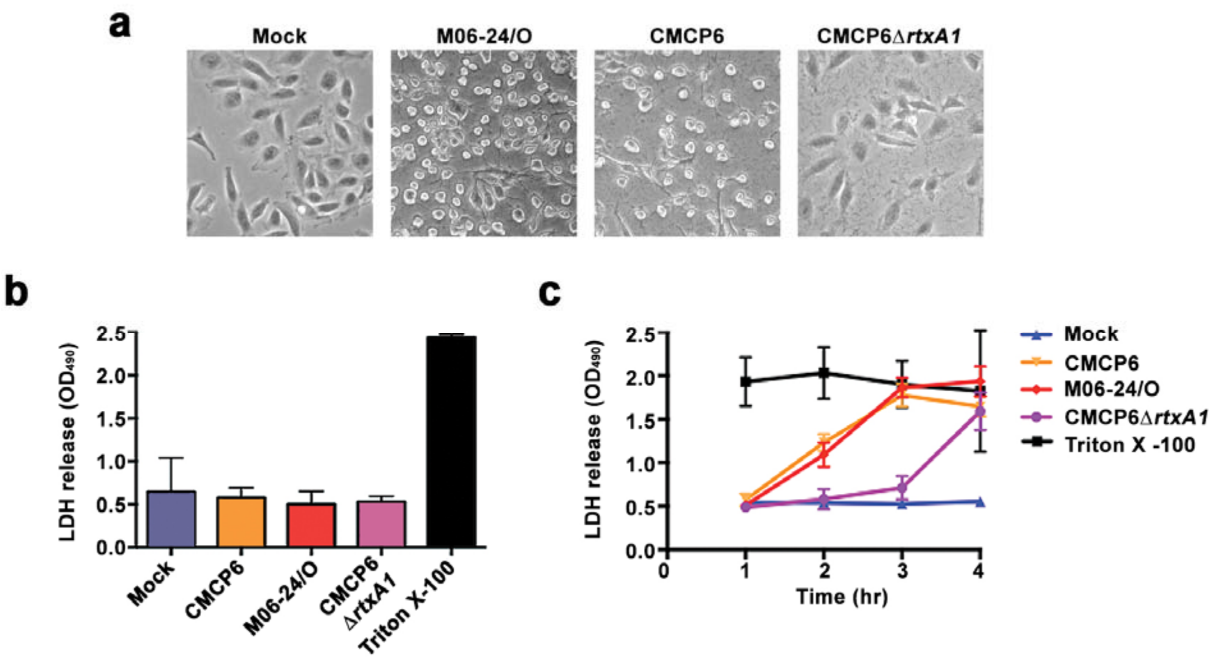

Supplementary Figure 7 │HeLa cell rounding and lysis due to *V. vulnificus*.

V. vulnificus MARTX toxins have distinct compositions dependent upon the strain isolate, as shown in Fig. 4a. Representative (*n*=3) phase images of cell rounding (**a**) and LDH release (**b**) induced after 60 min co-incubation of bacteria as indicated with HeLa cells, at which point cells were collected for detection of Ras and pERK in Fig.4b. (**c**) Cell lysis over time after addition of bacteria. Note that after 3 h, even bacteria without *rtxA1* induce cell lysis due to the *vvhA*-encoded cytolysin/hemolysin (Fan *et al*. *Infect Immun* **69**, 5943-5948 (2001)). Error bars represent mean ± standard deviation.

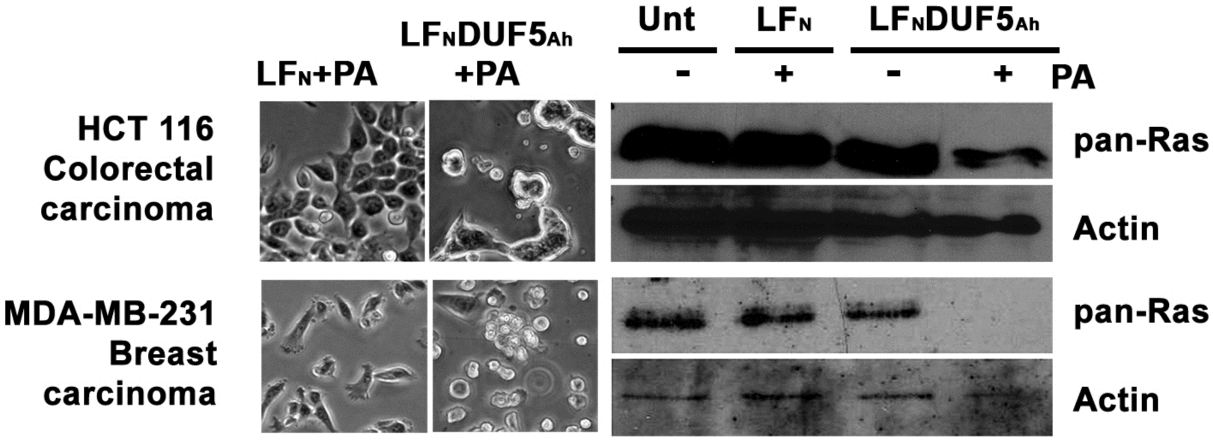

Supplementary Figure 8 │ Malignant cells are affected by DUF5$_{Ah}$ from *A. hydrophila.*
Phase images and immunoblot detection of Ras from HCT116 and MDA-MB-231 treated as indicated for 24 h.

Fig. 1b

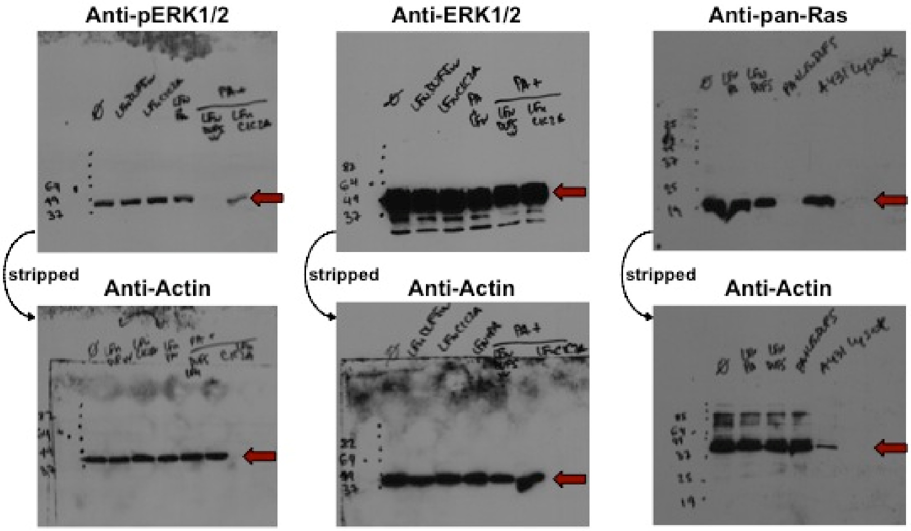

Fig. 1d

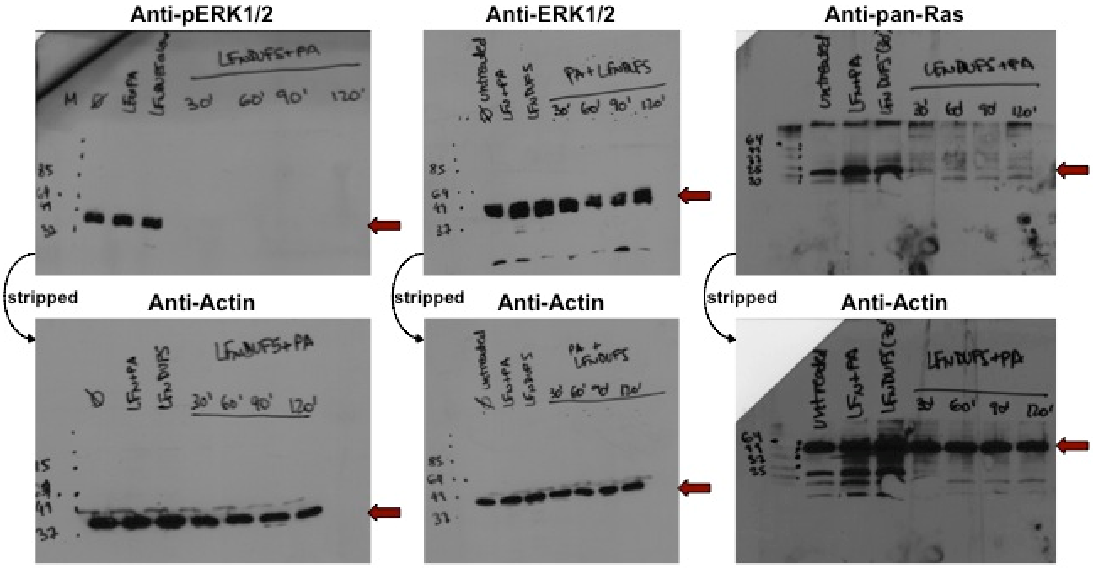

Supplementary Figure 9 │ Full size images of all western blots of Figure 1.

(1b) Full size images western blotting shown in Figure 1b. **(1d)** Full size images western blotting shown in Figure 1d. Red arrows indicate each expected proteins.

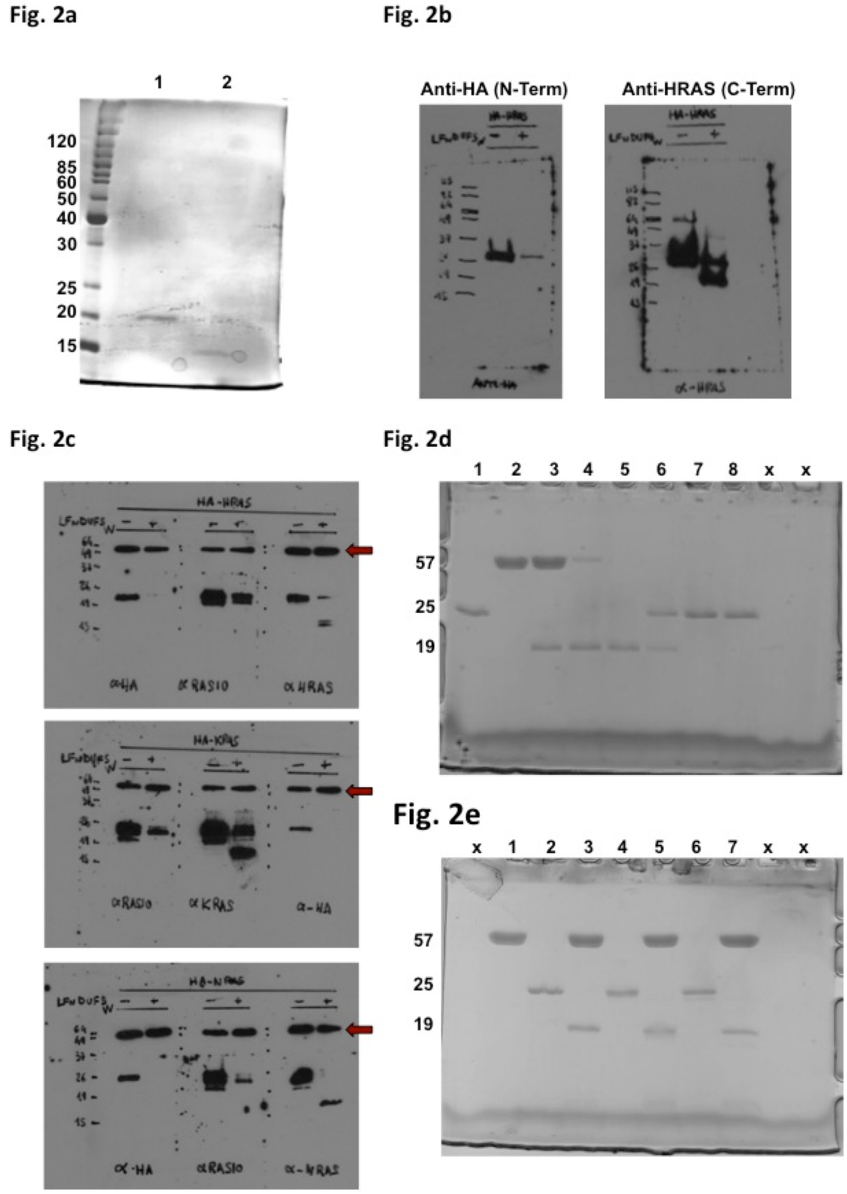

Supplementary Figure 10 │ Full size images of all western blots and gels of Figure 2.

(2a) Full size image of gel shown in Figure 2a, (1) untreated sample, (2) treated sample **(2b)** Full size images western blotting shown in Figure 2b. **(2c)** Full size images western blotting shown in Figure 2c, red arrows indicate tubulin. **(2d)** Full size image of gel shown in Figure 2d, (1) rKRas 10 μM, (2) rDUF5vv 10 μM, (3) rKRas/rDUF5vv [10/10] μM (4) rKRas/rDUF5vv [10/1] μM, (5) rKRas/rDUF5vv $[10/10^{-1}]$ μM, (6) rKRas/rDUF5vv $[10/10^{-2}]$ μM, (7) rKRas/rDUF5vv $[10/10^{-3}]$ μM, (8) rKRas/rDUF5vv $[10/10^{-4}]$ μM. X indicates empty lanes. **(2e)** Full size image of gel shown in Figure 2e, (1) rDUF5vv, (2) rKRas, (3) rDUF5vv and rKRas (4) rHRas (5) rDUF5vv and rHRas (6) rNRas, (7) rDUF5vv and rNRas.

Fig. 3b

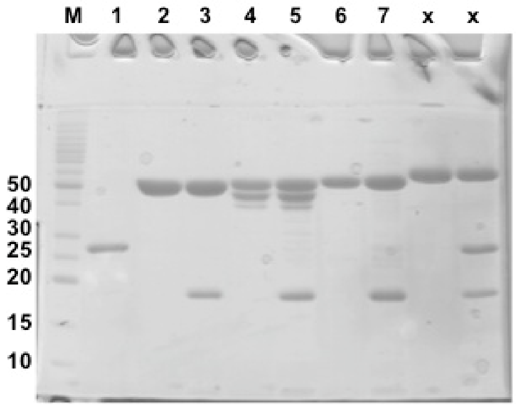

Fig. 3c

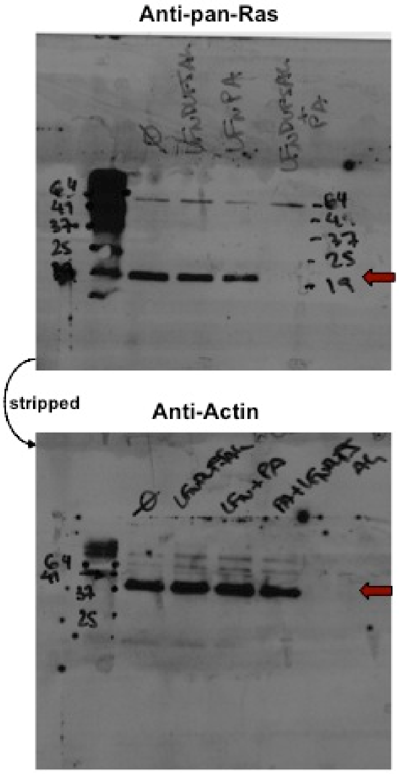

Fig. 3f

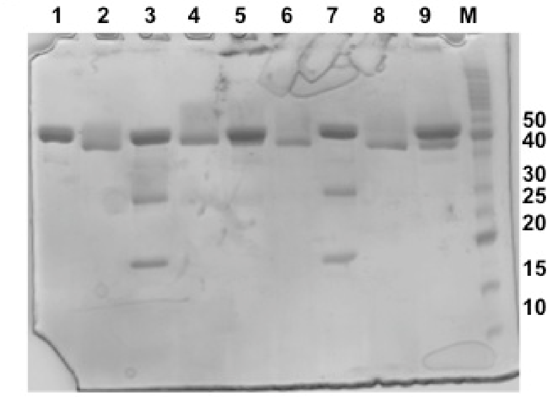

Supplementary Figure 11 │ Full size images of all western blots and gels of Figure 3. (3b) Full size image of gel shown in Figure 3b, (M) molecular weight ladder protein, X indicates unrelated experiments. **(3c)** Full size images western blotting shown in Figure 3c. Red arrows indicate each expected proteins. **(3f)** Full size image of gel shown in Figure 3f, (1) rDUF5vv, (2) GST-HRas, (3) rDUF5vv and GST-HRas (4) GST-RalA (5) rDUF5vv and GST-RalA, (6) GST-Rap1A, (7) rDUF5vv and GST-Rap1A, (8) GST-HRheb2, (9) rDUF5vv and GST-Rheb2, (M) molecular weight ladder protein.

Fig. 4b

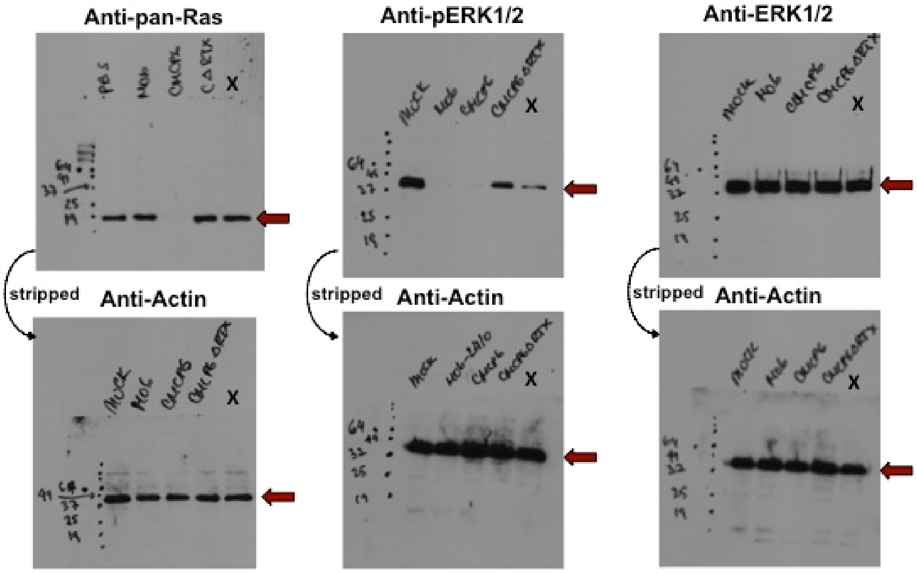

Fig. 4c

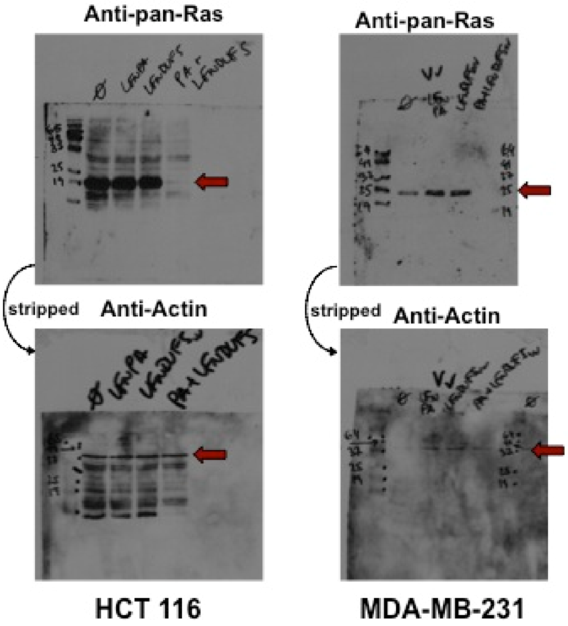

Fig. 4d

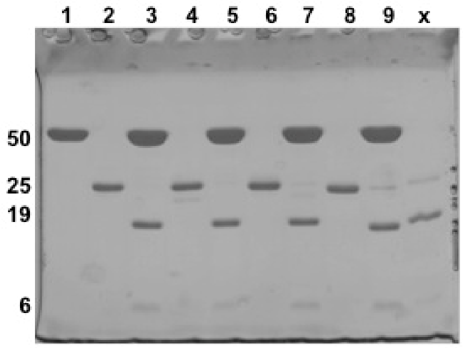

Supplementary Figure 12 │ Full size images of all western blots and gels of Figure 4. (4b) Full size image western blotting shown in Figure 4b. **(4c)** Full size image western blotting shown in Figure 4c. Red arrows indicate each expected proteins. X indicates unrelated experiments. **(4d)** X indicates unrelated experiments.

Sup. Fig. 2a

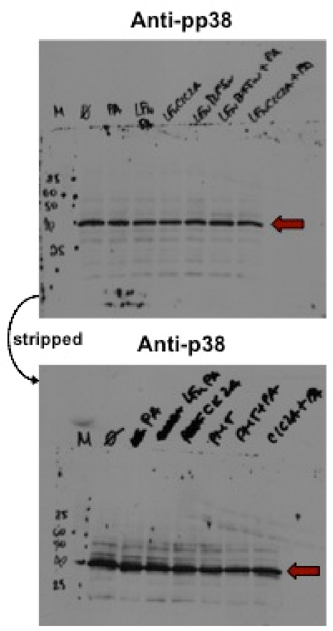

Sup. Fig. 2b

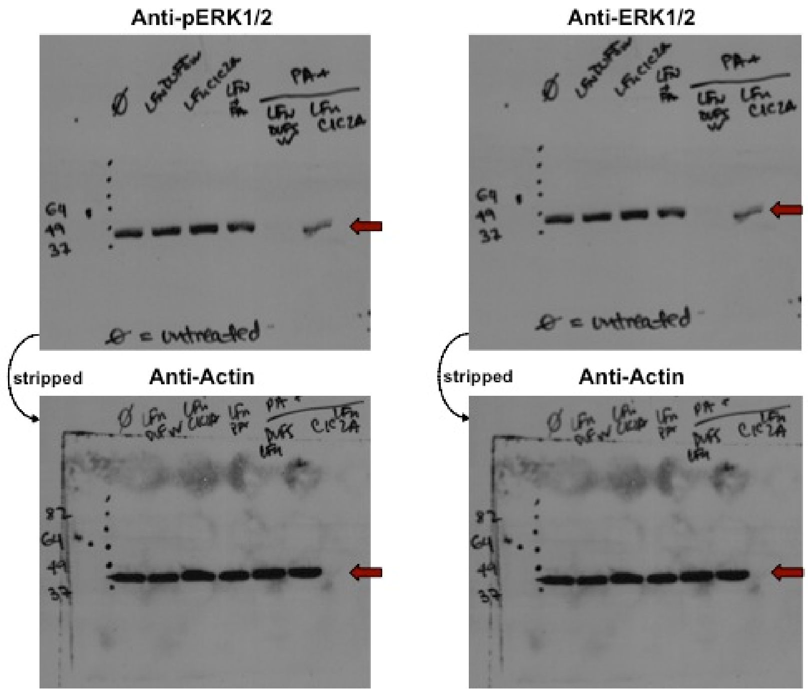

Supplementary Figure 13 │ Full size images of western blots for Supplementary Fig. 2. (2a) Full size image western blotting shown in Supplementary Figure 2a. **(2b)** Full size image western blotting shown in Supplementary Figure 2b. Red arrows indicate each expected proteins.

Sup. Fig. 4

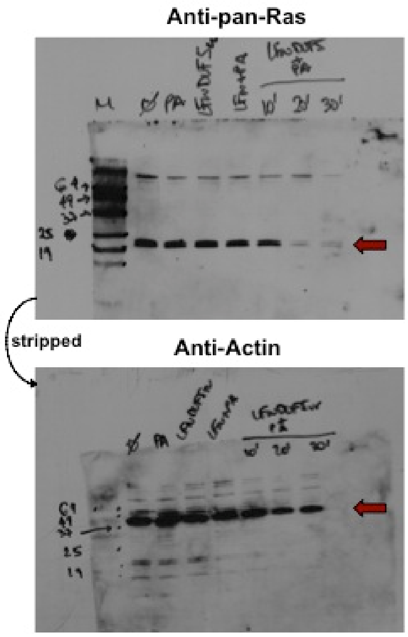

Supplementary Figure 14 │ Full size images of all western blots for Suppl. Fig. 4. (2a) Full size image western blotting shown in Supplementary Figure 4.

Fig. 3e / Sup. Fig. 5

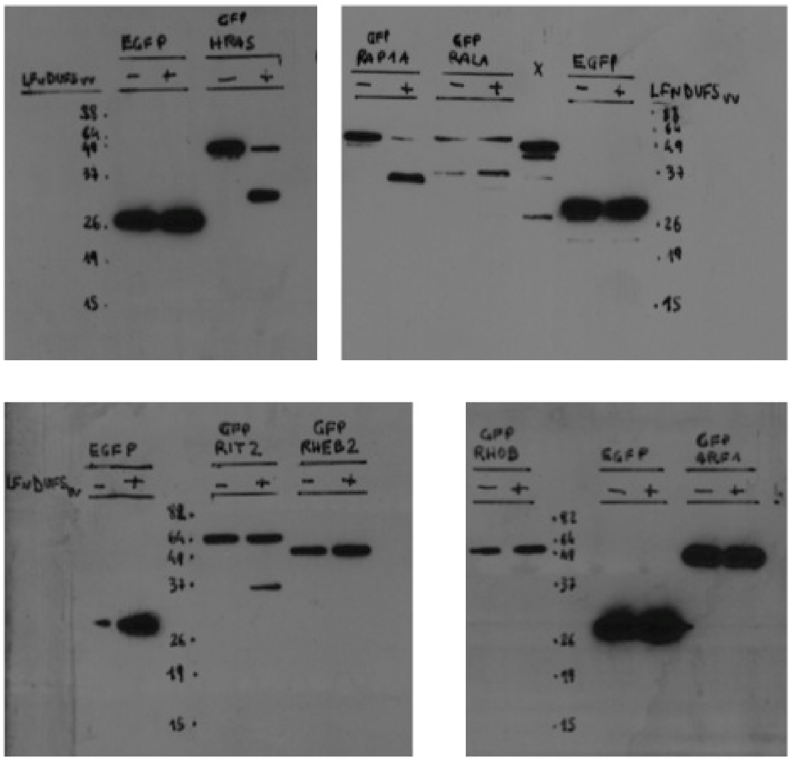

Supplementary Figure 15 │ Full size images of western blots for Suppl. Fig. 3e and 5.

Sup. Fig. 6

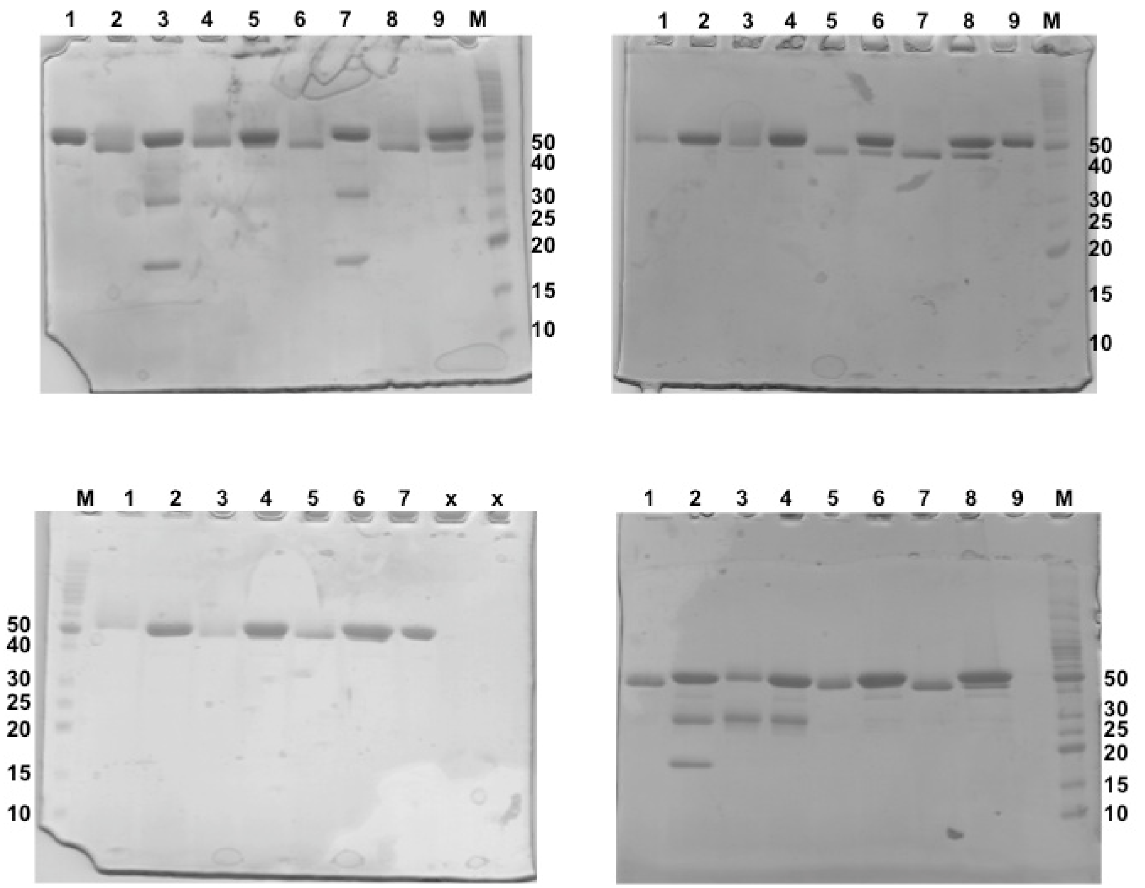

Supplementary Figure 16 │ Full size images of gels of Supplementary Figure 6.

(Top left corner) (1) rDUF5vv, (2) GST-HRas, (3) rDUF5vv and GST-HRas (4) GST-RalA (5) rDUF5vv and GST-RalA, (6) GST-Rap1A, (7) rDUF5vv and GST-Rap1A, (8) GST-Rheb2, (9) rDUF5vv and GST-Rheb2. **(Top right corner)** (1) GST-RhoA, (2) rDUF5vv and GST-RhoA, (3) GST-RhoB (4) rDUF5vv and GST-RhoB (5) GST-Rac1, (6) rDUF5vv and GST-Rac1, (7) GST-Cdc42, (8) rDUF5vv and GST-Cdc42, (9) rDUF5vv. **(Bottom left corner)** (1) GST-Rab4A, (2) rDUF5vv and GST-Rab4A, (3) GST-Rab11A (4) rDUF5vv and GST-Rab11A (5) GST-Ran, (6) rDUF5vv and GST-Ran, (7) rDUF5vv, X indicates unrelated experiments. **(Bottom right corner)** (1) GST-HRas, (2) rDUF5vv and GST-Ras, (3) GST-Rit2 (4) rDUF5vv and GST-Rit2 (5) GST-RalA, (6) rDUF5vv and GST-RalA, (7) GST-Rheb2, (8) rDUF5vv and GST-Rheb2, (9) rDUF5vv. (M) molecular weight ladder protein.

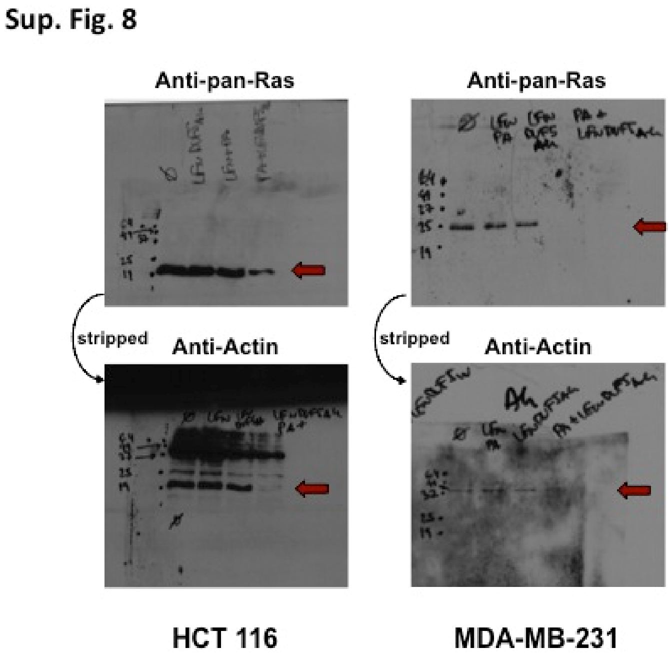

Supplementary Figure 17 │ Full size images of all western blots Supplementary Figure 8*.*
Red arrows indicate each expected proteins.

Supplementary Table 1. Oligonucleotides used in the study

DUF5 VV FWD	TACTTCCAATCCAATGCTCAAGAGCTGAAAGAAAGAGCAAAAG
DUF5 VV REV	TTATCCACTTCCAATGCTACAAACTGCCCTTGAACGTG
DUF5 AH FWD	TACTTCCAATCCAATGCTCCGGGCAAAACGGTGGTGACG
DUF5 AH REV	TTATCCACTTCCAATGCTAGACATCGGCGTACTCGACCCGC
DUF5 PA FWD	TACTTCCAATCCAATGCTCCATTACTCCATGACCTCATCACC
DUF5 PA REV	TTATCCACTTCCAATGCTACACATCATCATAACACTTGCG
KRAS FWD	TACTTCCAATCCAATGCTATGACTGAATATAAACTTGTGGTAGTTGGAGCTGG
KRAS REV	TTATCCACTTCCAATGCTACATAATTACACACTTTGTCTTTGACTTCTTTTTCTTC
HRAS FWD	TACTTCCAATCCAATGCTATGACGGAATATAAGCTGGTGGTGGTG
HRAS REV	TTATCCACTTCCAATGCTAGGAGAGCACACACTTGCAGCTC
NRAS FWD	TACTTCCAATCCAATGCTATGACTGAGTACAAACTGGTGGTGG
NRAS REV	TTATCCACTTCCAATGCTACATCACCACACATGGCAATCCC
EGFPC3-GST FWD	GCTTCGAATTCTGCACCCGGGTGGTCTGGTTCCGCGTGGA
EGFPC3-GST REV	CTAGATCCGGTGGATCCCCTCAGTGGTGGTGGTGGTGGTGC
KRAS_G13D FWD	TAGTTGGAGCTGGTGACGTAGGCAAGAGTGC
KRAS_G13D REV	GCACTCTTGCCTACGTCACCAGCTCCAACTA
KRAS_Q61R FWD	GATATTCTCGACACAGCAGGTAGAGAGGAGTACAGTGCAATG
KRAS_Q61R REV	CATTGCACTGTACTCCTCTCTACCTGCTGTGTCGAGAATATC
